# A rare mutation in hypophosphatasia: a case report of adult form and review of the literature

**DOI:** 10.20945/2359-3997000000108

**Published:** 2019-02-01

**Authors:** Francisco Galeano-Valle, Jaime Vengoechea, Rodolfo J. Galindo

**Affiliations:** 1 Hospital General Universitario Gregorio Marañón Hospital General Universitario Gregorio Marañón Department of Internal Medicine Madrid Spain Department of Internal Medicine, Hospital General Universitario Gregorio Marañón, Madrid, Spain; Instituto de investigación Sanitaria Gregorio Marañón, Madrid, Spain; Instituto de investigación Sanitaria Gregorio Marañón Madrid Spain; 2 Emory University School of Medicine Departments of Human Genetics and Medicine Atlanta GA USA Emory University School of Medicine, Departments of Human Genetics and Medicine, Atlanta, GA, USA; 3 Emory University School of Medicine Diabetes and Endocrinology Section Atlanta GA USA Emory University School of Medicine, Diabetes and Endocrinology Section, Atlanta, GA, USA

## Abstract

Hypophosphatasia is a rare inborn error of metabolism characterized by low serum alkaline phosphatase activity due to loss-of-function mutations in the gene encoding the tissue-nonspecific isoenzyme of alkaline phosphatase (TNSALP). Extracellular accumulation of TNSALP substrates leads to dento-osseous and arthritic complications featuring tooth loss, rickets or osteomalacia, and calcific arthopathies. Mild hypophosphatasia usually has autosomal dominant inheritance, severe cases are either autosomal recessive or due to a dominant negative effect. Clinical manifestations of hypophosphatasia are extremely variable, ranging from life threatening to asymptomatic clinical presentations. The clinical presentation of the adult-onset hypophosphatasia is highly variable. Fractures, joint complications of chondrocalcinosis, calcifying polyarthritis and multiple pains may reveal minor forms of the disease in adults. It is important to recognize the disease to provide the best supportive treatment and to prevent the use of anti-resorption drugs in these patients. Bone-targeted enzyme-replacement therapy (asfotase alfa) was approved in 2015 to treat pediatric-onset hypophosphatasia. We present a case of a 41-year-old male diagnosed with adult form of hypophosphatasia with a rare *ALPL* mutation that has been previously described only once and review the literature on the adult form of the disease and its genetic mechanism.

## INTRODUCTION

Hypophosphatasia (OMIM 146300, 241500, 241510) is a rare inborn error of metabolism characterized by low serum alkaline phosphatase (ALP) activity (hypophosphatasaemia) due to lossoffunction mutations within the gene that encodes the tissue-nonspecific isoenzyme of ALP (*ALPL*). In hypophosphatasia, extracellular accumulation of ALP substrates, including inorganic pyrophosphate, an inhibitor of mineralization, explains the dento-osseous and arthritic complications featuring tooth loss, rickets or osteomalacia, and calcific arthropathies (1). In mild hypophosphatasia, there is functional loss of one of the copies of the gene, leading to autosomal dominant inheritance. In severe hypophosphatasia, the mutations affect both alleles of the gene. This could be through autosomal recessive transmission or because of an autosomal dominant mutation with a dominant negative effect, in which the gene product from one allele interferes with homo-dimerization (2). The prevalence of severe and moderate hypophosphatasia forms were estimated at 1/300,000 and 1/6,370 patients in Europe, respectively (3). Clinical manifestations of hypophosphatasia are extremely variable and seven major clinical forms are identified. Fractures, joint complications of chondrocalcinosis, calcifying polyarthritis and multiple pains may reveal minor forms of the disease in adults. It is important to recognize the disease to provide the best treatment, to prevent fractures, which are frequently complicated and associated with pseudarthrosis. Moreover, it's extremely important to prevent the inappropriate use of anti-resorption drugs, frequently prescribed for the fractures related to bone fragility, and attributed to primary osteoporosis in these patients (1,4–6). Bone-targeted enzyme-replacement therapy with asfotase alfa was approved in 2015 to treat pediatric-onset hypophosphatasia. Currently, there is no well-established treatment available for the correction of the bone fragility in adult hypophosphatasia. We present a case of a 41-year-old male diagnosed with adult form of hypophosphatasia with a rare *ALPL* gene mutation that has been previously described only once and review the literature on the adult form of the disease and its genetic mechanism.

## CASE REPORT

**Figure 1:**
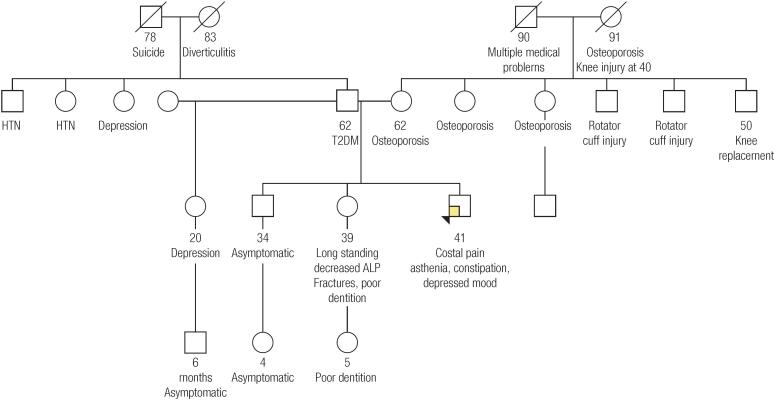
Pedigree of the patient's family. HTN: hypertension; ALP: alkaline phosphatase; T2DM: type 2 diabetes mellitus.

A 41-year-old Caucasian male presented to the endocrinology clinic with a long standing history of asthenia, constipation and depressed mood. Additionally, the patient related he had right lower ribs pain for several months. He denied any history of fractures, teeth loss, cavities, muscle pain, intellectual disability or vitamin supplementation. He had a family history of premenopausal osteoporosis in several family members, with poor response to bisphosphonates, and fractures. In addition, the patient had one sister with long-term decreased plasmatic ALP during adulthood, fractures and poor dentition (Figure 1). Physical examination revealed slight lack of enamel in teeth. Plasmatic calcium, phosphate, magnesium, thyroid and parathyroid hormone, kidney function, albumin and 25-hydroxyvitamin D (25(OH)D) were normal. Historical test results revealed decreased ALP for at least 2 years, ranging 18-20 U/L (normal range 45-115 U/L). Bone-specific ALP was 4.8 μg/L (normal range in males 6.5-20.1 μg/L). Vitamin B6 was 328.7 nmol/L (normal range 20-125 nmol/L). Vitamin A, B1, B2, B3, B5 and B12 were normal. Radiograph of the ribs did not reveal abnormalities. Kidneys ultrasound showed normal kidneys. After genetic counseling, *ALPL* gene (NM_000478.5) Sanger sequencing was performed at a CLIA-certified molecular diagnostic lab (Prevention Genetics, Marshfield, Wisconsin, USA). This showed that the patient was heterozygous for a pathogenic variant, c.1474del, which is predicted to result in a frameshift and premature protein termination (p.Ala492Profs*29). The classification as pathogenic was established by the clinical laboratory according to the 2015 ACMG/AMP guidelines. The diagnosis of autosomal dominant hypophosphatasia (adult form) was made. The patient was not enable to use enzyme replacement therapy (ERT) and the current treatment is basically symptomatic (analgesics, antidepressants), and focused on maintaining 25(OH)D within normal range and regular supervision by a multidisciplinary team including a dentist.

## DISCUSSION

The first case of hypophosphatasia was reported in 1948 by J. C. Rathbun (7) and the first identified *ALPL* mutation was reported in 1988 (8). In humans, four genes account for ALP. Three genes encode the tissue-specific intestinal, placental and germ-cell ALP, whereas the fourth gene, *ALPL*, encodes TNSALP, which is abundant in the skeleton, liver, kidney and developing teeth. TNSALP is actually a family of isoforms that differ by post-translational modifications. Homodimerization of TNSALP monomers is required for catalytic activity (9). ALPs circulate as soluble homodimers that are cleared by the liver (10). There is deficiency of all TNSALP isoforms in hypophosphatasia (9). The prevalence of severe and moderate hypophosphatasia forms were estimated at 1/300,000 and 1/6,370 respectively in the European population (3).

Three TNSALP phosphocompound substrates accumulate extracellulary in hypophosphatasia: both phosphoethanolamine (PEA) and inorganic pyrophosphate (PPi) in urine and blood and pyridoxal 5’-phosphate (PLP) in plasma which is the active form of vitamin B6 and a cofactor for several enzymatic reactions including neurotransmitters (1). PPi is a potent inhibitor of mineralization and its superabundance leads to the impairment of hydroxyapatite crystal formation and growth, thereby producing rickets and osteomalacia, respectively in children and adults, and a wide range of other symptoms including calcific arthropathies in some affected adults (4,6).

Hypophosphatasia shows an extraordinary range of severity that spans from death *in utero* with an unmineralized skeleton to dental complications or calcific periarthritis without bone disease in adulthood. Although its clinical spectrum is a continuum, hypophosphatasia has been classified into seven major clinical forms. Its outcome is conditioned principally by any skeletal complications, generally being worse with early life presentation (2,11,12).

Adult-onset hypophosphatasia typically presents during middle age (13). The main symptom is pain caused by fractures due to osteomalacia and PPi arthropathy, including pseudogout. The most frequent fractures involve the metatarsals. These fractures are recurrent, usually show a delayed consolidation and may lead to pseudarthrosis. The other characteristic fractures (or pseudofractures) affect laterally and proximally in the subtrochanteric femoral region (5,12–14). Dental abnormalities include enamel disorders, loose teeth and premature tooth loss. Calcific periarthritis, ossification of ligaments and nephrocalcinosis may be present. This form can become debilitating due to recurrent fracturing, skeletal and joint pain, and muscle pain. Recurring headaches and psychiatric symptoms (insomnia, anxiety, depression) are frequent (5,6,12–15).

Due to the extraordinary range of variability of the clinical features of the adult form, this is probably the most underdiagnosed form. Delayed diagnose is very common. A study showed that the onset of symptoms in adulthood occurred at a median age of 44 years and the median age at diagnosis was 49 years. At the time of presentation, one third of patients appeared asymptomatic (12).

Hypophosphatasia has been diagnosed traditionally when persistent hypophosphatasemia matches a medical history, physical examination, routine laboratory studies, and radiographic findings consistent with the diagnosis. The degree of hypophosphatasemia and TNSALP substrate accumulation generally reflects the severity of hypophosphatasia (1,6). TNSALP substrate excess in hypophosphatasia is marked with greatest sensitivity and specificity by elevated serum PLP (1,7). *ALPL* gene analysis is expected to reveal a defect in all patients with hypophosphatasia (2). Although persistent hypophosphatasaemia is the hallmark for the diagnosis of all forms of hypophosphatasia, it is not usually recognized in the clinical setting in hospitalized adult patients and it is rarely investigated further (16). Hypophosphatasaemia can also result from use of certain drugs and conditions (17). Assaying serum PLP is particularly helpful when hypophosphatasaemia has an explanation other than hypophosphatasia, because an elevated level might be expected exclusively in hypophosphatasia as the activities of all TNSALP isoforms, not just from bone, are low, and the other causes of hypophosphatasaemia seem to particularly suppress bone TNSALP activity (1,6). Documentation of an elevated PEA level in blood or urine supports a diagnosis of hypophosphatasia. However, PEA excretion can be unremarkable in mild hypophosphatasia (17). Currently, assays for PPi are carried out only in research laboratories (1). Radiological findings of adult hypophosphatasia include osteopenia, poorly-healing metatarsal stress fractures, pseudofractures, pyrophosphate arthropathy and calcific periarthritis (14,18,19). Z-scores assessed by DXA are only slightly reduced in most adults (1,14). Weak muscles appear normal on routine laboratory testing (20).

Although hypophosphatasia can typically be diagnosed without *ALPL* mutation analysis, this information is crucial for understanding inheritance. Hypophosphatasia derives from any mutation in the *ALPL* gene located on chromosome 1p36.1 that causes decreased TNSALP activity and increased levels of its substrates. All patients with hypophosphatasia carry one or two mutations involving *ALPL* (6). Three hundred and fifty one mutations have been identified and are reported in the *ALPL* gene mutation database (http://www.sesep.uvsq.fr/03_hypo_mutations.php, accessed on March 24, 2018) in North American, Japanese, and European patients, indicating a very strong allelic heterogeneity in the disease. This variety of mutations results in highly variable clinical expression and in a great number of compound heterozygous genotypes (21). The majority of the known mutations are missense point mutations with the remainder being composed of microlesions, splicing mutations, nonsense mutations, and a nucleotide substitution affecting the major transcription initiation site (17,22–24). The high clinical variability of the disease is correlated to the pattern of inheritance and depends on the large number of missense mutations and their variable effect on TNSALP activity. Autosomal dominant and autosomal recessive transmission of these defects generally explain mild versus severe hypophosphatasia, respectively. Missense mutations that interfere with the formation of the TNASLP dimer can decrease enzymatic function considerably and result in a dominant negative effect, accounting for cases of autosomal dominant perinatal hypophosphatasia. Significantly different phenotypic presentations of the disease may even occur within a family (2,15).

Despite our patient having mild and unspecific symptoms, persistently low ALP levels and the strong family history of bone and joint problems lead to the suspicion of hypophosphatasia, that was confirmed by genetic testing. Genetic counseling was performed to inform possible affected relatives. This patient was heterozygous in exon 12 of the *ALPL* gene for a variant defined as c.1474del, which is predicted to result in a frameshift and premature protein termination (p.Ala492Profs*29) and therefore the diagnosis of autosomal dominant hypophosphatasia (adult form) was made. This variant, along with another *ALPL* variant, has been reported by Brun-Heath and cols. (the report uses a non-current reference transcript so the variant is described as c.1471del) in a patient with perinatal lethal hypophosphatasia (24). In addition, this variant has been only observed in 1 of 236,966 alleles in a public database, indicating it is rare (http://gnomad.broadinstitute.org/variant/1-21904036-CG-C). All the reported deletions result in a frameshift with early termination and mRNA decay or significantly shorter proteins. In that reported case, the mutation only affected the last 30 amino acids of the protein, a region where no missense mutation had ever been described, however the fetus was affected with perinatal lethal hypophosphatasia. *In vitro* experiments performed using a protein with a deletion of the last 30 amino acids showed that in the absence of this region, the protein was not excreted but retained a normal enzymatic activity. This indicates that, because the deletion occurs in the last exon of the gene, it escaped mRNA decay, and this region was necessary for membrane anchoring but not for enzymatic activity (25). The lethal phenotype observed for that patient suggested that membrane anchoring is essential for TNSALP function and confirmed the importance of the carboxy-terminal part of the protein. Finally, as previously reported (26), the mutations resulting in deletions and insertions resulting in frameshift are responsible for the more severe forms of hypophosphatasia, due to the aberrant product of the gene (24). We performed molecular analysis of the *ALPL* gene only, it is possible that a modifier variant elsewhere in the genome could be a contributor to the patient's phenotype.

Management of adult patients with hypophosphatasia must include assessment of bone and joint complications, in addition to chronic pain and mood disorders (5). Antiresorptive therapies are not recommended, as they may worsen underlying osteomalacia (27). Currently, there is no established treatment available for the correction of the bone fragility in adult hypophosphatasia. Bone-targeted ERT (asfotase alfa) was approved in 2015 to treat pediatric-onset hypophosphatasia. However, the adult form management is typically supportive (6,28). Recombinant PTH was tried on various clinical cases and it had positive results. In a clinical trial, asfotase alfa improved functional outcomes in some adolescents and adults with hypophosphatasia. However, the data is still limited to make strong recommendations (5).

In conclusion, Hypophosphatasia is a rare inborn error of metabolism with a wide range of clinical features. Adult form accounts for the most common and the least severe form and it is often difficult to be recognized with a delayed diagnosis and inappropriate treatments. *ALPL* mutation testing is being increasingly used in clinical practice, confirming the need to manage mutation findings by thorough clinical examination to distinguish hypophosphatasia patients with subclinical manifestations from those with biochemical abnormalities but who are otherwise asymptomatic. The mutation c1474del has been previously described only once in medical literature.
